# Spectrum of genes involved in a unique case of Potocki Schaffer syndrome with a large chromosome 11 deletion 

**DOI:** 10.5414/NP300691

**Published:** 2014-05-05

**Authors:** Bernd F.M. Romeike, Yiping Shen, Hiromi Koso Nishimoto, Cynthia C. Morton, Lawrence C. Layman, Hyung-Goo Kim

**Affiliations:** 1 Institute of Pathology, Neuropathology Section, Jena University Hospital, Friedrich-Schiller-University, Jena,; 2 Institute of Neuropathology, Saarland University, School of Medicine, Homburg/S., Germany,; 3 Genetic Diagnostic Laboratory, Department of Laboratory Medicine, Children’s Hospital Boston, Waltham, MA,; 4 Department of Obstetrics and Gynecology, Institute of Molecular Medicine and Genetics, Georgia Regents University, Augusta, GA, and; 5 Departments of Obstetrics, Gynecology, and Reproductive Biology and of Pathology, Brigham and Women’s Hospital and Harvard Medical School, Boston, MA, USA

**Keywords:** Potocki Schaffer syndrome

## Abstract

Letter to the Editor.

Sir, – A unique case of Potocki Schaffer syndrome (PSS) was reported previously [1], but at that time the diagnosis could not be genetically confirmed. Here, we delineate more detailed neuropathological findings, present a detailed list of deleted genes confirmed by array comparative genomic hybridization (aCGH), and discuss possible genotype/phenotype relations in this patient. 

In short, in addition to obligate exostoses (EXT) and parietal foramen (FPP), the patient also suffered from severe intellectual disability (ID). Motor development was also retarded with documented regression of motor function at the age of 3. After puberty, athetotic movements and seizures developed. In later life, he suffered from recurrent respiratory tract infections, anemia, thrombocytopenia, and finally generalized edema. He never walked alone, he was non-verbal and unable to perform activities of daily living. He manifested craniofacial anomalies (CFA) including a high and broad forehead, brachycephaly, a long narrow pointed nose, strabismus, thin palpebral fissures, the absence of eyebrows, large dysplastic low set ears, and a hypoplastic mandible (Figure 1A). At various hospital admissions, obesity, cryptorchidism, over stretchable joints, contractures, osteoporosis, hip dysplasia, and hypertrophic cardiomyopathy were noted. Shortly before death, a cranial computerized tomography (CCT) scan at the age of 33 demonstrated, in addition to micrencephaly, severe brain atrophy with hydrocephalus e vacuo, a large cavum Vergae, and choroid plexus arachnoid cyst in the occipital horn of the left side ventricle as well as hyperostosis frontalis (Figure 1B). These findings were also confirmed by autopsy on coronary slices of formalin-fixed brain sections (Figure 1C). 

Detailed histomorphological study of FPP revealed associated lipomatous tissue in the center (Figure 1D). Bony rims of FPP demonstrated dense predominantly lamellar bone. Despite rather large defects histomorphologically, FPP demonstrated remarkable preservation of bony lamellae, representing extreme thinning rather than true loss of bone (not shown). The hyperostosis frontalis spared the area of the sagittal sinus (Figure 1E). Histomorphologically, the bone tissue was predominantly composed of lamellar bone, and the bone marrow contained fat tissue and hematopoietic cells (Figure 1F), and osteoclasts were infrequent and only observed with CD68 immunohistochemistry (Figure 1F inset). In the occipital horn of the left ventricle, an arachnoid cyst of the choroid plexus was found (Figure 1G). Histology revealed large cysts lined by arachnoid cells (Figure 1H). The surrounding tissue contained macrophages and psammoma bodies. At the surface, little pre-existing choroid plexus was preserved. In the right ventricle, a choroid plexus cholesteatoma was found in the occipital horn (Figure 1I). Histology showed a typical cholesteatoma with numerous cholesterol crystal clefts, macrophages, multinuclear giant cells of foreign body type, and siderophages in the fibrous stroma as well as some psammoma bodies (Figure 1J). At autopsy, the detailed neuropathologic examination of the micrencephalic brain revealed evidence of two separate diseases. First, a disturbance of mass growth of neurons with micrencephaly, periventricular nodular heterotopias, a small degree of cortical dysplasia of the cerebellum, sometimes grouped nodular heterotopias, and, not mentioned in the previous report, single ectopic ganglion cells were scattered in the subcortical white matter of the cerebral hemispheres. The second central nervous system (CNS) disease included symmetric accumulations of macrophages at certain sites in the white matter and cortex as described earlier [1]. These findings resembled a metabolic storage disease and fit well with the clinically described decline of motor function. 

For the array CGH, DNA was extracted from formalin fixed and paraffin embedded brain tissue of the patient. In short, an Agilent 244K human genome oligonucleotide CGH microarray (G4411B) was used for array CGH analysis following the manufacturer’s instructions. Images were captured by an Agilent scanner at the resolution of 2 µm and quantified using Feature Extraction software v9.0 (Agilent Technologies, Palo Alto, CA, USA). CGH analytics software v3.4 (Agilent Technologies, Palo Alto, CA, USA) was subsequently used for data normalization, quality evaluation, and data visualization. Copy number variants (CNV) were called using the ADM-2 (Aberration Detection Method 2) algorithm. A minimum of five probes was required to meet the size cut-off for a CNV. The average inter-probe spacing is ~ 8.9 Kb, thus the average CNV detection sensitivity is ~ 45 Kb. 

This study revealed an 11.9 Mb deletion at 11p between 38,824,655 and 50,638,770 (hg18). The karyotype was confirmed as del(11)(p11.12p12). An OMIM database search revealed more than 50 annotated genes in this region (Table 1). 

Included are ALX4, which explains FPP, EXT2 accounting for multiple exostoses, and the MYBPC3 gene involved in hypertrophic cardiomyopathy (HCM) of the patient. Thus, not only the PSS was confirmed, but also that the patient is the first to be described with documented hypertrophic cardiomyopathy (HCM). Diagnosis of HCM during his lifetime was probably possible because he is the oldest one so far reported with PSS permitting development of a cardiomyopathy. Furthermore, all genes postulated to be deleted in the first report were validated in addition to others [1]. 

Besides FPP and EXT, for which the genes ALX4 and EXT2 are well established, osteoporosis and hyperostosis frontalis were additional features observed in this patient. The simultaneous occurrence of bone loss, such as osteoporosis and FPP, and gain of bone material at other sites, i.e., EXT and hyperostosis frontalis, is quite remarkable. A possible explanation could be related to selective vulnerability/susceptibility of different bones at different sites and different gene expression status. Different types of ossification or osteogenesis of bones, i.e., intramembranous vs. enchondral ossification, might explain these differences. The parietal bones ossify intramembranously from ossification centers that appear during the 8^th^ and 9^th^ weeks of fetal life. From the literature, it is known that there is a size reduction of FPP with advancing age (mechanical or disclosure type defect), but there can also be a more prominent intracranial phenotype in consecutive generations [2]. 

Indeed, the patient described here is the first one with PSS with detailed pathologic macroscopic and microscopic findings. The more detailed histomorphological findings of FPP described here reveal that the true bony defect resulted from extreme thinning of the parietal bone. Because there are no vessels or any other structures entering or exiting the cranial vault at this site, the terms “hereditary bi-parietal diminished/delayed ossification”, “parietal bone hypoplasia”, or “thinning of the parietal bone” might actually better characterize the underlying pathology. The origin of the lipomatous tissue at this site remains obscure, but a malformative genesis appears most likely. As described in our previous report, osteoporosis might be associated with ACP2 [1]. 

A candidate gene for hyperostosis frontalis would be SPI1 as failure to express the gene product might preclude development of osteoclasts, leading to arrested bone resorption and osteopetrosis [3]. Indeed, our detailed histomorphological search of osteoclasts in the area of hyperostosis frontalis revealed relatively few small osteoclasts that were only detectable by means of immunohistochemistry. Hyperplasia of calvarial bones was also described as a CCT finding by Wuyts et al. [4]. 

The candidate region for intellectual disability (ID) in PSS was previously localized to 11p11.2 between D11S1361 and D11S1344, which is the same region postulated for craniofacial abnormalities (CFA) [5, 6]. PHF21A was identified after the candidate gene region was narrowed to 1.1 Mb by deletion mapping and after PHF21A was found to be disrupted in 3 unrelated balanced translocation patients with ID and craniofacial anomalies [7]. Furthermore, the work of Wuyts and colleagues points to a possible additional locus in the region telomeric to EXT2 [4]. Apart from PHF21A responsible for ID and CFA [7], several other candidate genes for both phenotypes include PEX16 and ACP2, whereas ID alone could be related to SLC35C1, MDK, AMBRA1, ARHGAP1, MADD, or FOLH1. Multiple loci for ID are further supported by the phenotype. Two different diseases, a CNS malformation and a metabolic disease, imply two different genes for ID. 

The brain malformations including micrencephaly, ventriculomegaly i.e., hydrocephalus e vacuo, cavum Vergae, nodular heterotopias, and cerebellar cortical dysplasia, which is now supplemented by scattered ectopic ganglion cells, are obviously due to disturbed growth of neurons. In the previous report [1], two candidate genes were identified: SLC35C1 and MDK. As delineated in Table 1, there are now some additional candidate genes expressed in the brain that are involved in apoptosis, migration, or neural development. These additional genes include API5, AMBRA1, ARHGAP1, and MADD. 

To date, no other PSS patient with an additional metabolic storage disease has been described. Also, regression of motor function was not clinically documented in other reported PSS patients. Thus, this phenotype might constitute a new and separate syndrome. The candidate gene for metabolic disease is ACP2, which constitutes a separate ID locus because it is located in a centromeric direction from the generally postulated ID locus in PSS between D11S1361 and D11S1344. 

For the first time, the detailed macroscopic and microscopic findings of a choroid plexus cholesteatoma and choroid plexus arachnoid cyst of a PSS patient are reported. The cholesteatoma might be a sequelae due to high amounts of fatty acids. Genes with influence on fatty acid metabolism include HSD17B12, MAPK8IP1, NR1H3 and FOLH1, all of which might also be responsible for obesity and consequent morbidities. A choroid plexus cyst seems to be a recurrent finding of PSS as it has been described in 1 patient (PSS12) in the series of Wakui et al. [5] and in a recent series in 2 of 6 patients studied with magnetic resonance imaging (MRI) [8]. 

Despite several imaging studies, patients with nodular heterotopias have not been reported previously [4, 5, 8, 9, 10, 11, 12]. CT might not provide sufficient resolution, but MRI might be able to delineate these lesions. 

In conclusion, the previously described unique clinical description of a patient with PSS was confirmed by array CGH. This male is the oldest PSS patient described so far, the only singular individual with hypertrophic cardiomyopathy linked to deletion of MYBPC3, and the first autopsied with detailed neuropathologic studies. The unambiguous presence of two different CNS diseases suggests that there are at least two loci or genes for intellectual disability in PSS. This assumption is also supported by the clinical course of the patient with early intellectual disability and later regression and development of athetotic movements and seizures. Arachnoid cysts of the choroid plexus are recurrent findings of PSS. Detailed histomorphological studies of the FPP revealed extreme thinning and hypoplasia of the parietal bone resembling hereditary bi-parietal diminished/delayed ossification rather than true foramina of the parietal bone. 

## Acknowledgments 

We thank the family of the person whose details are described here for their permission to perform an autopsy, publish the findings, and providing photographs of the patient; B. Kramann Institute for Radiodiagnostics, University of the Saarland, Homburg Saar, Germany, is gratefully acknowledged for providing the CCT. 

Funded by grant from NIH GM061354 (CCM). 

## Conflict of interest 

The authors declare no conflict of interest. 

**Table 1.** Genes involved in sequence of their physical position retrieved from: www.ncbi.nlm.nih.gov/omim/gene (accessed in March 2013).

Gene/OMIM	Name	Phenotype or function: questionable (?), hypothetical (°) or likely (*) role in PSS
*LRRC4C/608817*	Leucine-rich repeat-containing protein 4C	Promotes outgrowth of thalamocortical axons?
*API5/609774*	Apoptosis inhibitor 5	Expression prevents apoptosis?
*TTC17*	Tetratricopeptide repeat domain 17	Unknown?
*HSD17B12/609574*	17-beta-hydroxysteroid dehydrogenase XII	Fatty acid elongation?
*ALKBH3/610603*	AlkB, E. coli, homolog of,3	Repair of single-stranded DNA lesions?
*ACCS/608405*	1-aminocyclopropane-1-carboxylate synthase	Catalyzes the deamination of L-vinylglycine?
EXT2/608210	**Exostosin 2**	Loss of activity causes hereditary multiple osteochondroma (exostoses)* ^p^
ALX4/605420	**Aristaless-like 4, mouse, homolog of**	Deletion or mutation results in foramina parietalia permagna* ^p^ **, polydactyly°**
*CD82/600623*	CD 82 antigen, formerly: KAI1	Metastasis suppressor gene, activation of T-cells?
*TSPAN18*	Tetraspanin 18 isoform 1	Unknown?
*TP53I11*	Tumor protein p53-induced protein	Unknown?
*PRDM11*	PR domain containing 11	Unknown?
*SYT13/607716*	Synaptotagmin 13	Vesicular traffic, exocytosis, and secretion, neurotransmitter release?
*CHST1/603797*	Carbohydrate sulfotransferase1	Corneal transparency, macular corneal dystrophy?
*SLC35C1/605881*	Solute carrier family 35, member C1, formerly: FUCT1	Congenital disorder of glycosylation type IIc with immunodeficiency°^p^ and severe mental and growth retardation°^p^, brain malformations°^p^, seizures°^p^, hypotonia°^p^, recurrent infections°^p^, bleeding disorder°^p^
*CRY2/603732*	Cryptochrome 2	Regulator of circadian feedback loop?
*MAPK8IP1/604641*	Mitogen-activated protein kinase 8-interacting protein 1	Non-insulin-dependent diabetes mellitus°^p^, adipositas°^p^, CNS damage°^p^, genitourinary abnormalities°^p^
*PEX16/603360*	Peroxisome Biogenesis Factor 16	Zellweger syndrome, peroxisomal disorder – candidate? for craniofacial anomalies°^p^, mental and growth retardation°^p^, hypotonia°^p^, seizures°^p^
*GYLTL1B/609709*	Glycosyltransferase-like 1B	Unknown?
PHF21A/608325	**PHD finger protein 21A**	Repression of neuron-specific genes - causative for intellectual disability and craniofacial anomalies* ^p^ ** [9]**
*CREB3L1*	CAMP responsive element binding protein 3-like 1	Transcription factor; acts during endoplasmic reticulum stress by activating unfolded protein response target genes?
*DGKZ/601441*	Diacylglycerol kinase, zeta	T-cell regulation?
*MDK/162096*	Midkine, formerly NEGF2	Angiogenesis, cell growth, and cell migration – candidate for brain malformation°^p^, mental retardation°^p^, seizures°^p^
*CHRM4/118495*	Cholinergic receptor, muscarinic, 4	Unknown?
*AMBRA1/611359*	Activating molecule in beclin 1-regulated autophagy	Neural development – candidate for brain malformation with nodular heterotopias°^p^
*HARBI1/615086*	Harbinger transposase derived 1	Transposase-derived protein that may have nuclease activity (potential). Does not have transposase activity?
*ATG13/615088*	Autophagy-related protein 13	Unknown?
*ARHGAP1/602732*	Rho GTPase-activating protein 1	Cell migration – candidate for brain malformation with nodular heterotopias°^p^
*ZNF408*	Zinc finger protein 408	May be involved in transcriptional regulation?
*F2/176930*	Coagulation factor II	Hypo-, dys-, or hyper-prothrombinemia, thromboses, hemorrhages; bleeding disorder°^p^
*CKAP5/611142*	Cytoskeleton-associated protein 5	Microtubule organization?
*LRP4/604270*	Low density lipoprotein receptor-related protein 4	Sclerosteosis 2, brachydactyly or syndactyly. Associated with Cenani-Lenz syndactyly syndrome and Mulefoot disease?
*C11orf49*	Chromosome 11 open reading frame 49	Interacts with uranyl acetate?
*ARFGAP2/606908*	ADP-ribosylation factor GTPase-activating protein 2	Unknown?
*PACSIN3/606513*	Protein kinase C and casein kinase substrate in neurons 3	Vesicle formation and transport?
*DDB2/600811*	DNA damage-binding protein 2	Repair of DNA damage induced by UV radiation; xeroderma pigmentosum groupe E?
*ACP2/171650*	Acid phosphatase 2, lysosomal	Lysosomal storage°, mental and growth retardation °^p^, seizures°^p^, hypotonia°^p^, osteoporosis°^p^, hyperostosis frontalis°^p^, craniofacial dysostosis°^p^, bleeding disorder°^p^, edemas°^p^
*NR1H3/602423*	Nuclear receptor subfamily 1, group H, member 3	Lipid homeostasis, cholesterol accumulation in peripheral tissues, adipositas°^p^, cardiovascular disease, reduced inflammation or macrophage function.
*MADD/603584*	MAP kinase-activating death domain	Prevents apoptotic signaling – candidate for brain malformation with nodular heterotopias°^p^
*MYBPC3/600958*	Myosin-bindig protein C, cardiac	Cardiomyopathy*^p^
*SPI1/165170*	Spleen focus forming virus proviral integration oncogene SPI1, aka PU.1	Transcription factor of hematopoietic cells, arrested bone resorption and osteopetrosis, hyperostosis frontalis°^p^
*SLC39A13/608735*	Solute carrier family 39 (zinc transporter), member 13	Skeletal and dental abnormalities. Ehlers-Danlos syndrome-like spondylocheirodysplasia?
*PSMC3/186852*	Proteasome 26S subunit, ATPase, 3	Tat-mediated transcriptional activation?
*RAPSN/601592*	Receptor-associated protein of the synapse, 43-KD	Myasthenia, fetal akinesia syndrome?
*CUGBP1/601074*	CUG triplet repeat, RNA-binding protein 1	RNA-binding protein implicated in the regulation of several post-transcriptional events; pre-mRNA alternative splicing; mRNA translation and stability?
*PTPMT1/609538 *	Protein-tyrosine phosphatase, mitochondrial, 1	Protein phosphatase; specifically mediates dephosphorylation of mitochondrial proteins, thereby playing an essential role in ATP production?
*KBTBD4*	Kelch repeat and BTB (POZ) domain containing 4	Interacts with acetaminophen?
*NDUFS3/603846*	NADH-ubiquinone oxido-reductase Fe-S protein 3	Complex I, mitochondrial respiratory chain defect; Leigh syndrome?
*FAM180B*	Family with sequence similarity 180, member B	Unknown?
*C1QTNF4/614911*	Complement C1q and tumor necrosis factor related protein 4	Unknown?
*MTCH2/613221*	Mitochondrial carrier homolog 2	Mitochondrial protein?
*AGBL2*	ATP/GTP binding protein-like 2	Cytoplasmic metallocarboxypeptidase; may play a role in regulation of microtubuli organization?
*FNBP4/615265*	Formin binding protein 4	Binds FMN1. Interacts with the Arg/Gly-rich-flanked Pro-rich of KHDRBS1/SAM68?
*NUP160/607614*	Nucleoporin, 160-KD	Mediation of RNA export from the nucleus?
*PTPRJ/600925*	Protein-tyrosin phosphatase, receptor-type, J	Susceptibility to (colorectal) cancer?
*OR4B1, * *OR4X2, * *OR4X1, * *OR4S1, * *OR4C3, * *OR4C45, * *OR4A47*	Olfactory receptor, family 4	Interact with odorant molecules in the nose, to initiate a neuronal response that triggers the perception of a smell?
*FOLH1/600934*	Folate hydrolase 1	Impaired intestinal absorption of dietary folates with consequent hyperhomocysteinemia with increased risk for cardiovascular disease – adipositas°p, neural tube defects, and cognitive deficits°p; glutamate excitotoxicity.

Top to bottom equals telomere to centromere direction; ^p^symptoms and signs present in the patient described here.

**Figure 1.**
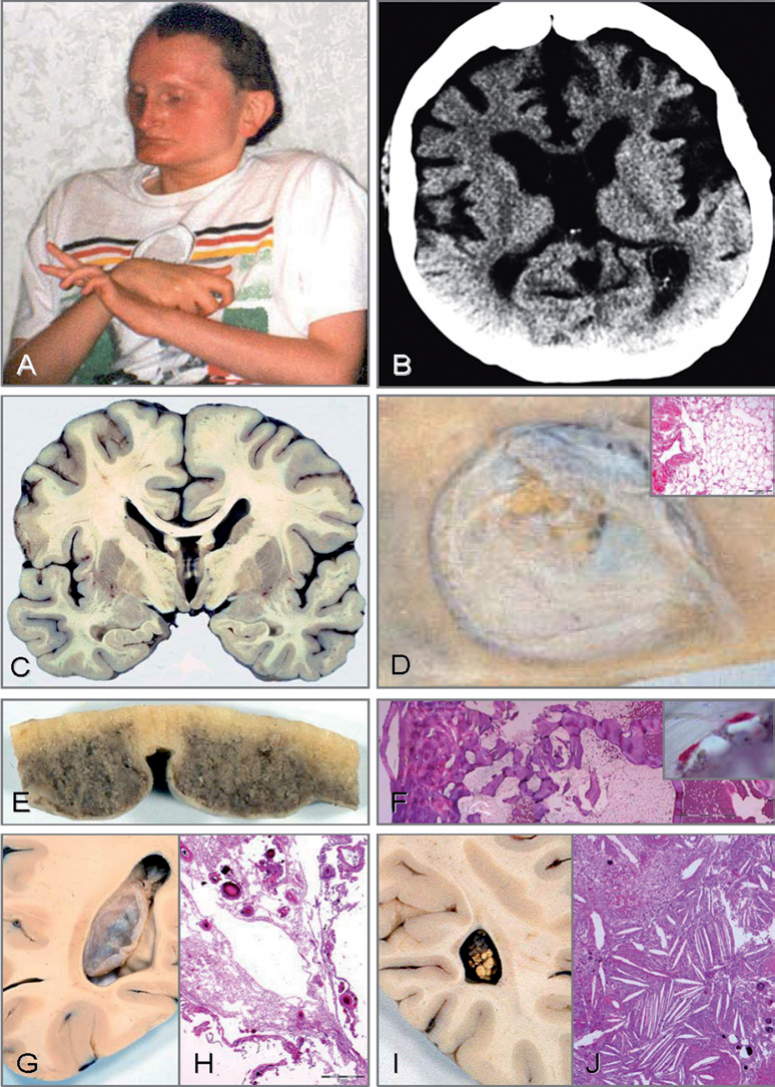
PSS phenotype, CCT, macroscopic findings, and histopathology. The patient at age 33 years (A); a CCT obtained shortly before death shows micrencephaly, severe brain atrophy with hydrocephalus evacuo, large cavum Vergae, and choroid plexus arachnoid cyst (B); coronal slice of formalin fixed brain at autopsy with large cavum Vergae and evacuo hydrocephalus (C); view of FPP from the internal aspect showing yellow fat tissue at the center (D), inset shows histology of the yellowish material disclosing lipomatous tissue, hematoxylin-eosin stain, scale bar 200 µm; formalin fixed frontal bone with severe hyperostosis (E); transection of frontal bone (F), the outside is left, the epidural space at the right, bone marrow contains fat tissue and hematopoiesis, hematoxylin-eosin stain in polarized light, scale bar 2 mm, inset shows immunohistochemistry for CD68 indicating few small osteoclasts, alkaline phosphatase, scale bar 20 µm; arachnoid cyst of the choroid plexus of the left occipital horn (G); histology reveals large cysts lined by arachnoid cells, the surrounding contains macrophages and psammoma bodies, at the outer rims little pre-existing choroid plexus is delineated, hematoxylin-eosin stain, scale bar 1 mm (H); in the right occipital horn, the choroid plexus contained a cholesteatoma (I), typical histomorphological appearance of cholesteatoma with numerous cholesterol crystal clefts, macrophages, multinuclear giant cells of foreign body type, siderophages, in the background fibrous stroma, and some psammoma bodies, hematoxylin-eosin stain, scale bar 500 µm (J).
